# Molecular Survey of Vector-Borne Pathogens of Dogs and Cats in Two Regions of Saudi Arabia

**DOI:** 10.3390/pathogens10010025

**Published:** 2020-12-31

**Authors:** Abdullah D. Alanazi, Abdulaziz S. Alouffi, Mohamed S. Alyousif, Mohammad Y. Alshahrani, Hend H. A. M. Abdullah, Sobhy Abdel-Shafy, Nichola Eliza Davies Calvani, Maryam Ansari-Lari, Alireza Sazmand, Domenico Otranto

**Affiliations:** 1 Department of Biological Sciences, Faculty of Science and Humanities, Shaqra University, P.O. Box 1040, Ad-Dawadimi 11911, Saudi Arabia; 2 King Abdulaziz City for Science and Technology, Riyadh 12354, Saudi Arabia; asn1950r@gmail.com; 3 Department of Zoology, College of Science, King Saud University, P.O. Box 2455, Riyadh 11451, Saudi Arabia; msaifi@ksu.edu.sa; 4 Department of Clinical Laboratory Sciences, College of Applied Medical Science, King Khalid University, P.O. Box 61413, Abha 9088, Saudi Arabia; moyahya@kku.edu.sa; 5 Department of Parasitology and Animal Diseases, Veterinary Research Division, National Research Centre, 33 Bohouth St., Dokki, Giza 12622, Egypt; vet_nrc_2006@yahoo.com (H.H.A.M.A.); aasobhy@yahoo.com (S.A.-S.); 6 Veterinary Pathology Diagnostic Services, Sydney School of Veterinary Science, Faculty of Science, The University of Sydney, Sydney, NSW 2006, Australia; nichola.calvani@sydney.edu.au; 7 Department of Food Hygiene and Public Health, School of Veterinary Medicine, Shiraz University, Shiraz 7144169155, Iran; ansari@shirazu.ac.ir; 8 Department of Pathobiology, Faculty of Veterinary Science, Bu-Ali Sina University, Hamedan 6517658978, Iran; domenico.otranto@uniba.it; 9 Zoonotic Diseases Research Center, School of Public Health, Shahid Sadoughi University of Medical Sciences, Yazd 8915173160, Iran; 10 Department of Veterinary Medicine, University of Bari, 70010 Bari, Italy

**Keywords:** *Anaplasma*, *Babesia*, *Bartonella*, *Mycoplasma*, one health, Saudi Arabia, vector-borne pathogens, zoonosis

## Abstract

Dogs and cats play an important role as reservoirs of vector-borne pathogens, yet reports of canine and feline vector-borne diseases in Saudi Arabia are scarce. Blood samples were collected from 188 free-roaming dogs and cats in Asir (70 dogs and 44 cats) and Riyadh (74 dogs), Saudi Arabia. The presence of *Anaplasma* spp., *Bartonella* spp., hemotropic *Mycoplasma* spp., *Babesia* spp., and *Hepatozoon* spp. was detected using a multiplex tandem real-time PCR. PCR-positive samples were further examined with specific conventional and real-time PCR followed by sequencing. Dogs from Riyadh tested negative for all pathogens, while 46 out of 70 dogs (65.7%) and 17 out of 44 cats (38.6%) from Asir were positive for at least one pathogen. Positive dogs were infected with *Anaplasma platys* (57.1%), *Babesia vogeli* (30%), *Mycoplasma haemocanis* (15.7%), and *Bartonella henselae* (1.4%), and cats were infected with *Mycoplasma haemofelis* (13.6%), *Candidatus* Mycoplasma haemominutum (13.6%), *B. henselae* (9.2%), and *A. platys* (2.27%), all of which are reported for the first time in Saudi Arabia. Co-infection with *A. platys* and *B. vogeli* was detected in 17 dogs (24.28%), while coinfections were not detected in cats. These results suggest that effective control and public awareness strategies for minimizing infection in animals are necessary.

## 1. Introduction

Vector-borne pathogens (VBPs) transmitted by ticks, fleas, and mosquitoes, are of global importance especially in the case of zoonotic infections which pose a direct threat to human health and animal welfare [1,2,3]. Vector-borne diseases (VBDs) as a result of infection with viral, bacterial, and protozoal VBPs are often widespread in tropical and subtropical regions [4], including in the Middle East, due to the optimal climatic conditions for the perpetuation of arthropods involved in their transmission [5]. In this context, vector-borne infections of dogs and cats—that have a close relationship with humans in both urban and rural areas—pose potential public health concerns [6]. However, data on the occurrence of canine and feline VBDs are only available in a few countries of the Middle East such as Qatar [7], Iraq [8], and Iran [9]. In a previous study from the Riyadh Province, 37% of dogs’ blood DNA samples (20/53) were suspected positive for *B. vogeli*, *Mycoplasma haemocanis*, and *Candidatus* Mycoplasma haematoparvum, yet none were confirmed using Sanger sequencing [10]. Unlike elsewhere in Saudi Arabia, Riyadh is a semi-arid area with low annual rainfall and relative humidity, making it unsuitable for tick species that prefer more temperate or tropical climates, such as *Rhipicephalus sanguineus* (Latreille, 1806) sensu lato (s.l.). While much of the country is similarly hot and dry, the coastal areas of Saudi Arabia and those at higher altitudes may be more accommodating to parasites capable of VBD transmission. Investigations into ectoparasite and VBP prevalence in these regions, however, remain lacking, which makes the impact of VBDs on animal and human populations difficult to quantify.

Scant scientific information is available regarding the tick fauna of dogs and cats in Saudi Arabia and the pathogens they harbor. Two tick species, *Rh. sanguineus* s.l. and *Hyalomma dromedarii* (Koch, 1844), commonly infest dogs in Saudi Arabia [10,11]. In regard to cats only *Rh. sanguineus* s.l. and *H. dromedarii* have been reported in Saudi Arabia [12]. Both tick species are known to be vectors and/or intermediate hosts of a variety of parasites, bacteria, and viruses, some of which are zoonotic agents [13]. For instance, *Rh. sanguineus* s.l. plays an important role in the transmission of numerous pathogens, including members of the genera *Babesia*, *Hepatozoon*, *Anaplasma*, *Ehrlichia*, *Rickettsia*, and hemotropic mycoplasmas [14,15]. *Hyalomma dromedarii* is also known to be a vector of *Rickettsia aeschlimannii*, *Rickettsia africae*, and the protozoan parasite *Theileria annulata* [16]. 

Likewise, there are no data on the fleas of dogs and cats in Saudi Arabia and the flea-borne infections in these animals. Fleas are vectors and intermediate hosts of various bacteria (e.g., hemotropic *Mycoplasma* spp., *Bartonella* spp., *Rickettsia* spp.) and parasites, e.g., *Acanthocheilonema reconditum* and *Dipylidium caninum* [17]. The cat flea *Ctenocephalides felis* (Bouche, 1835), one of the main vectors of *Bartonella henselae*—the causative agent of the “cat scratch disease”—is the predominant flea species found on cats and dogs worldwide [18]. Both *C. felis* [19] and human infection with *B*. *henselae* have been reported in Saudi Arabia [20]. However, there is no information about the prevalence of bartonellosis in dogs and cats in this country. 

In previous studies from the Riyadh Province, the tested dogs’ blood DNA samples were found infected with VBPs [10]; and 49.5% of dogs and 13.8% of cats were infested with ticks, identified as *H. dromedarii* and *Rh. sanguineus* s.l. [10,11], the latter of which is a competent vector of several vector-borne pathogens [3,21]. Hence, the aim of the current study was to add to the existing knowledge on the identity, prevalence, and distribution of VBPs in Saudi Arabia by screening free roaming dogs and cats from two locations, one temperate and one semi-arid (Riyadh and Asir Provinces, respectively).

## 2. Results

### 2.1. Presence of VBPs in Dogs 

Multiplex tandem real-time PCR (MT-PCR) revealed that dogs from the Riyadh Province tested negative for all the pathogens; however, 46 out of 70 dogs (65.7%) from the Asir Province were PCR-positive for at least one of the tested pathogens, i.e., *Anaplasma* spp., *Babesia* spp., *Bartonella* spp., and *Mycoplasma* spp. Specific PCRs showed that dogs (Table 1) were mainly infected with *A. platys* (57.1%), followed by *B. vogeli* (30.0%), *M. haemocanis* (15.7%), and *B. henselae* (1.4%). Co-infection with *A. platys* and *B. vogeli* was detected in 17 dogs (24.3%). 

The nucleotide sequences of *A. platys* isolate from a dog in Asir (accession number MW199129) was 100% identical to *A. platys* of dogs from China (MN630836), South Africa (MK814413–MK814421), and Cuba (KX792089) and from *Armigeres subalbatus* (Culicidae) from China (KU586175). The sequence of one *B. vogeli* (accession number MW204836) was 100% identical to *B. vogeli* of dogs from Malawi (LC556376–LC556377), China (MK881089) and from cats in China (MN067708–MN067709). The *Mycoplasma haemocanis* sequence (accession number MW280824) was 100% identical to isolates from dogs in South Korea (MT345534), Mexico (MN294708), Chile (KY117659), Taiwan (KJ858513), Thailand (KU765208), Brazil (KP715860) and cats in Brazil (KM275242, KM275246–KM275247). 

### 2.2. Presence of VBPs in Cats 

In total, 17 out of the 44 cats (38.6%) tested positive according to the MT-PCR, with a higher prevalence of *M. haemofelis*/*M. haemocanis* (13.6%) and *Candidatus* Mycoplasma haemominutum (13.6%), followed by *B. henselae* (9.1%) and *A. platys* (2.3%) (Table 1). 

Nucleotide sequences of one *B. henselae* isolate from a cat (accession number MW208843) was >99% identical to *B. henselae* isolates from small Indian mongoose (*Herpestes auropunctatus*) in Grenada, West Indies (MG680299, MG680304, MG680305, MG680309, MG680313).

Nucleotide sequences of *M. haemofelis* from two cats (accession numbers MW280822–MW280823) were 100% identical to *M. haemofelis* isolates from cats in Brazil (KM275242, KM275246–KM275247) and one single-nucleotide polymorphism (SNP) different from the *M. haemofelis* strain Ohio2 (CP002808), also identical with *M. haemocanis* isolates from dogs in Iran (KU886264) and dogs in Turkey (KX641903) and *Pulex irritans* in Argentina (MK097143). 

### 2.3. Risk Factor Analysis in Dogs

Summary statistics for the animals’ characteristics and risk factors are presented in Table 2. The presence of *A. platys* DNA was significantly associated with age, with more one-year-old dogs (78.9%, 15/19) considered PCR-positive than older age groups (49%, 25/51) (OR = 3.9, *p*-value = 0.03). In contrast to *A. platys*, the presence of *M. haemocanis* DNA was significantly higher in dogs older than 4-years-old (34.5%, 10/29) when compared to younger animals (2.4%, 1/41). Results of the multivariable binary logistic regression analysis showed that dogs older than 4-years-old were 18.6 times more likely to be PCR-positive for *M. haemocanis* than dogs 3-years-old and younger (OR = 18.6, *p*-value = 0.009); and symptomatic dogs were 8.3 times more likely to be PCR-positive than asymptomatic ones (OR = 8.28, *p*-value = 0.038).

When the presence of DNA from any VBPs was considered as an outcome variable, male dogs (76.3%, 29/38) were more prone to being infected in comparison with female dogs (53.1%, 17/32). Odds of infection were 2.8 times higher in male dogs compared to females (OR = 2.8, *p*-value = 0.045). In addition, dogs positive for *M. haemocanis* DNA were more often symptomatic (36.4%, 4/11) than those PCR-negative (5.1%, 3/59). No other significant association was observed between infection with other hemoparasites and gender, health status, and infestation with ectoparasites.

### 2.4. Risk Factor Analysis in Cats 

The only significant association in cats was between health status and infection with *Candidatus* Mycoplasma haemominutum. Three out of a total of six infected cats were symptomatic compared with two of the 38 uninfected ones (OR = 18, *p*-value = 0.008). No significant association was detected between infection with VBPs in cats and the studied risk factors including sex, age, health status, and infestation with ectoparasites. 

### 2.5. Symptoms Associated with Pathogens

In 6/7 dogs and 3/5 cats that were PCR-positive for VBPs, fever, anorexia, emaciation, colics, and red eyes were observed. One dog and two cats with one or more symptoms, however, were PCR-negative for all the VBPs tested (Supplementary Table S1).

## 3. Discussion

The high occurrence of VBD-causing pathogens in free-roaming dogs (65.7%) and cats (38.6%) in the Asir Province indicates that animal populations are exposed to multiple VBPs, including those of zoonotic importance, posing both a companion animal and a public health risk. In comparison, however, the absence of all the pathogens in the dogs from Riyadh Province suggests that the climate is less conducive to the survival of ticks compared to the Asir Province, which may in turn contribute to a reduction in the transmission of VBDs. The absence of VBPs in the dogs from Riyadh in the current study is similar to the findings of a previous study, where 53 blood DNA samples from dogs in Riyadh were ultimately considered free from specific pathogen DNA [10]. The lack of VBPs in the previous study was attributed to the low prevalence of *Rh. sanguineus* s.l. parasitising dogs in Riyadh, likely due to its arid climate. Lower numbers of ticks were observed in Riyadh in the current study, providing further evidence that the low annual rainfall and relative humidity restricts the distribution of ectoparasites and the pathogens they vector in Riyadh.

In the Middle East, little information is available about the prevalence of canine and feline VBDs; however, results of previous studies in Qatar, Iraq, and Iran showed that stray dogs and cats often act as reservoirs of zoonotic pathogens with the prevalence of 18.8%, 38.1%, 54.6% in dogs [7,8,22] and 20.6%, 39.1% in cats [7,8] from Qatar, Iraq, and Iran. The prevalence of the *A. platys* infection in the dogs (57.1%) from the Asir Province is much higher in the present study than in the previous reports from the Middle East, e.g., 1.6% in Qatar [7], 3.33% in Iraq [8,23], 3.67% in Iran [22], 0.5% in Turkey [24], and 9.63% in Palestine [25]. Camels have also been found to be infected with *A. platys* in Saudi Arabia, making it the most prevalent tick-borne pathogen in the region [26] and suggesting that other mammalian hosts might play role in the epizootology of this parasite. In dogs, this infection is usually mild or asymptomatic, but animals may develop clinical signs such as anorexia, weight loss, lymphadenomegaly, hyperthermia, and hemorrhage, which can be fatal [27]. Not much is known, however, about the *A. platys* infection in cats. In two reports, naturally-infected cats exhibited anorexia, apathy, anuria, constipation, and jaundice [28,29]. Our statistical analyses revealed that infection with *A. platys* was more common in younger dogs, although breed, age, and sex have all been identified as risk factors for *A. platys* infections [30]. Although tick-borne transmission of *A. platys* remains the main route of circulation of this parasite, vertical transmission of *A. platys* from pregnant dogs to their offspring [31,32] suggests that controlling this infection in free-roaming dog populations might not be easy. 

In this study, hemoplasmas were detected in 27.2% of cats (13.6%, *M. haemofelis*, and 13.6%, *Candidatus* Mycoplasma haemominutum) and 15.7% of dogs (*M. haemocanis*). The above prevalence is higher than in the previous reports from Qatar, i.e., 2.9% for *M. haemofelis*, 2.9% for *Candidatus* Mycoplasma haemominutum, and 5.9% for *Mycoplasma* spp. in cats and 7.8% for *Mycoplasma* spp. in dogs [7]. However, the prevalence of hemoplasmosis in Saudi Arabia as determined in the current study is similar to that recorded in Iran, where 22–25.4% cats and 6.25–22% of dogs with single or multiple hemoplasmas have been reported [33,34,35,36,37]. Statistical analyses revealed that older dogs were infected more often than younger ones. It has been documented that other than the older age, cross-breeding, and mange infection, presence of vectors, bite wounds, neoplastic diseases, and rural vs. urban localities are risk factors for hemoplasmosis in dogs and cats [38,39]. Since hemotropic *Mycoplasma* spp. including *Candidatus* Mycoplasma haematoparvum can infect humans [40,41] and arthropods are considered major players in the epidemiology of hemoplasmas, control of fleas and ticks on dogs and cats is advocated. 

*Bartonella henselae* is herein reported for the first time in 9.1% of cats and 1.4% of dogs from Saudi Arabia. There is not much known about the epidemiology of bartonellosis in the Middle East. In Iran, infection of 7.14–12.5% of cats with *B. henselae* [42,43,44] and 12.5–24.24% of dogs with *Bartonella rochalimae*, *Candidatus* Bartonella merieuxii and *Bartonella vinsonii* subsp. *berkhoffii* has been confirmed by molecular analyses [9,45]. Overall seroprevalence of 74.2% for *B. henselae*, *Bartonella clarridgeiae*, and *B. vinsonii* subsp. *berkhoffii* was recorded in dogs [9]. Similarly, in Iraq, a seroprevalence of 15% for *B. henselae* and 12.6% for *B. clarridgeiae* in stray cats [46] and an overall seroprevalence of 47.4% in dogs for *B. henselae*, *B. clarridgeiae*, *B. vinsonii* subsp. *berkhoffii*, and *Bartonella bovis* was reported [47]. In Saudi Arabia, bartonellosis has been reported in 68% of Balochistan jirds (*Gerbillus nanus*), 60.33% of Libyan jirds (*Meriones libycus*), 13.39% of desert hedgehogs (*Paraechinus aethiopicus*), and one human patient [20,48,49]. Considering that in cats and dogs, the asymptomatic course with persistent bacteremia is a frequent outcome of infection [50], infected animals may pose high infection risks to owners or veterinary healthcare workers in endemic areas. 

The prevalence of *B. vogeli*-infected dogs (i.e., 29.9%) is higher than previously reported from Middle Eastern countries, e.g., 3.2% in Qatar [7], 1.5% in Palestine [51], 0.1% in Turkey [24], 0% in Iraq [8], and 1.13% in different regions of Iran [22]. Although *B. vogeli* and *Babesia canis* are known to infect cats [52], we could not detect DNA of either *Babesia* species in the blood of cats. Further molecular studies are needed to better understand canine and feline babesiosis in Saudi Arabia because the MT-PCR used to screen samples in the current study could only detect *B. vogeli* and *Babesia gibsoni*.

Dogs were not examined for *Ehrlichia canis* and *Anaplasma phagocytophilum*, the causative agents of monocytic ehrlichiosis and granulocytic anaplasmosis in dogs and humans [3,21]. While canine monocytic ehrlichiosis (CME) is a worldwide distributed tick-borne infection, *A. phagocytophilum* is more common in Europe and North America [30]. In a previous study from an eastern region of Saudi Arabia, CME was diagnosed by serology in nine dogs from a colony, while only one dog was PCR-positive [53]. In another study from Saudi Arabia, DNA from the blood of 53 dogs living in Riyadh tested negative for *E. canis* [10]. There are few reports on the prevalence of *E. canis*-infected dogs from other Middle Eastern countries, e.g., 2/64 in Qatar [7], 9/240 and 0/354 in Iran [22,54], 0/97 in Iraq [8]. Similarly, *A. phagocytophilum* does not seem to be prevalent in dogs of the region since in previous studies from Saudi Arabia, Qatar, Iraq, and five regions of Iran, the infection could not be detected [7,8,10,22]. However, DNA of *A. phagocytophilum* was found in the blood of camels from Riyadh [55]. Further studies are needed to determine domestic and wild reservoir hosts for *E. canis* and *A. phagocytophilum* in this country [3] as well as the tick species acting as vectors. However, the small number of samples examined in the current study prevent wider conclusions to be drawn regarding the prevalence of VBPs in Saudi Arabia. 

## 4. Materials and Methods 

### 4.1. Study Area and Sampling

**Figure 1:**
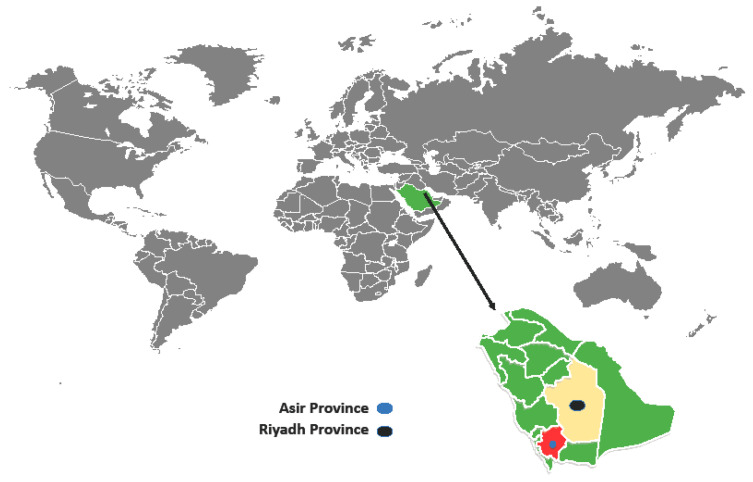
Map showing the study sites of the Riyadh Province and the Asir Province, Saudi Arabia.

The investigation was conducted from November 2018 to August 2019 in the Asir (19.0969° N, 42.8638° E) and Riyadh (24.7136° N, 46.6753° E) Provinces, located in southwestern and central Saudi Arabia, respectively (Figure 1). The Asir Province borders the Red Sea and Yemen, has an area of 76,690 km^2^, and is situated on a high plateau that receives more rainfall than the rest of the country and contains the country’s highest peaks, which rise to almost 3000 m above sea level. According to the Köppen-Geiger climate classification, this province has different climates, but the most prevalent ones are BWh (hot desert climates) and BSk (cold semi-arid climates). The average annual rainfall in the highlands ranges from 300 to 500 mm across two rainy seasons. As a result, there is much more natural vegetation and forests. Riyadh region’s climate, however, is BWh with virtually no rainfall during the year (http://www.pme.gov.sa).

### 4.2. Sampling of Dogs and Cats and Blood Collections

A total of 188 free-roaming dogs and cats in two provinces of Asir (70 dogs and 44 cats) and Riyadh (74 dogs) (Table 2) were trapped with a live animal trap (Havahart, Lancaster, PA, USA). Treats were provided for animals in the trap and they were restrained after having calmed down. From each animal, 0.5–3 mL blood samples were collected from the cephalic or saphenous vein in vacutainer tubes containing an EDTA anticoagulant (BD Vacutainer^®^ Tube, Gribbles Veterinary Pathology, Clayton, Victoria, Australia). Dogs and cats were set free after the blood collection. Animal data including age estimated by examination of teeth (1–6 years) and sex were recorded. All of the animals except seven dogs and five cats were apparently healthy. The symptoms of the sick animals were moderate or high fever, anorexia, emaciation, colics, and reddish eyes, though they were general and not exclusively related to the specific conditions caused by VBD-causing pathogens. Infestation of the animals with ticks, fleas, and lice was also recorded. Blood samples were refrigerated and transported to the Laboratory of Parasitology, Department of Biological Sciences, Faculty of Science and Humanities, Shaqra University.

### 4.3. DNA Extraction

Total genomic DNA (gDNA) was isolated from the blood samples using a Wizard^®^ Genomic DNA Purification Kit (Promega, Madison, WI, USA) and eluted into 50 μL or 100 μL of the elution buffer according to the manufacturer’s instructions. Aliquots of DNA were sent to the Veterinary Pathology Diagnostic Services (VPDS), Sydney School of Veterinary Science, The University of Sydney for molecular diagnostic processing and pathogen identification. Upon arrival at the VPDS, gDNA was stored at −20 ˚C for up to 1 month prior to screening.

### 4.4. Screening of VBPs by Multiplex Tandem Real-Time PCR (MT-PCR)

The diagnostics of VBPs was performed using the AusDiagnostics real-time quantitative MT-PCR panel for small animals’ anemia (Mascot, New South Wales, Australia) on the AusDiagnostics Easy-Plex^TM^ platform (Mascot, New South Wales, Australia). This automated two-step nested PCR simultaneously detects *A. platys*, *B. vogeli*, *B. gibsoni*, *Bartonella* spp., *Mycoplasma haemofelis*, *M. haemocanis*, *Candidatus* Mycoplasma haemominutum, and *Candidatus* Mycoplasma haematoparvum [56]. The assay was run using a 10 μL undiluted gDNA sample and each pathogen was detected during a short (10 cycles) conventional PCR (cPCR) followed by a second longer SYBR-based qPCR. 

### 4.5. Specific Pathogens’ Detection and Sequencing

The most positive samples for *Babesia* spp. (5 dogs), *Anaplasma* spp. (5 dogs), and *Mycoplasma* spp. (5 dogs and 5 cats) in addition to all the positive samples for *Bartonella* (4 cats and 1 dog) were further examined specifically by either cPCR using primers PLATYSF (5′-GATTTTTGTCGTAGCTTGCTATG-3′) and EHR16SR (5′-TAGCACTCATCGTTTACAGC-3′) for *A. platys* [28], HBT-F (5′-ATACGGCCCATATTCCTACG-3′) and HBT-R (5′-TGCTCCACCACTTGTTCA-3′) for *Mycoplasma* spp. [57], PIRO-A (5′-AATACCCAATCCTGACACAGGG-3′) and PIRO-B (5′-TTAAATACGAATGCCCC-3′) for *Babesia* spp. [58], or a multiplex TaqMan probe real-time PCR targeting the *ssrA* gene of *Bartonella* spp. with primers ssrA-F (5′-GCTATGGTAATAAATGGACAATGAAATAA-3′), ssrA-R (5′-GCTTCTGTTGCCAGGTG-3′), and 6-carboxyfluorescein (FAM)-labeled probe (5′-ACCCCGCTTAAACCTGCGACG-3′-BHQ1) [59]. All the cPCR reactions were run using the MyTaq^TM^ Red Mix (Bioline, Rhodes, Australia) in a Veriti^TM^ thermal cycler (Thermo Fisher Scientific, North Ryde, Australia) and real-time PCR reactions were run using a SensiFAST^TM^ Probe No-ROX Kit (Meridian Bioscience, Eveleigh, Australia) that does not need a passive reference signal of carboxy-X-rhodamine (ROX) for normalization of the data on a CFX95 Touch^TM^ Real-Time PCR detection system (Meridian Bioscience, Eveleigh, Australia). A negative control and a positive control were included in each run. 

All PCR products were separated by electrophoresis in 2% agarose gel stained with GelRed^TM^ (Biotium, Fremont, CA, USA) and visualized using UV light. Samples that displayed discrete bands of expected sizes were sent to Macrogen Ltd. in Seoul, South Korea, for bidirectional sequencing using amplification primers. Unambiguous sequences were assembled and compared to the closely related NCBI GenBank sequences using CLC Main Workbench ver. 6.8.1 (CLC bio, Vedbæk, Denmark). Representative sequences of pathogens detected in this study were deposited in GenBank (Table 1).

### 4.6. Statistical Analysis

Statistical analyses were performed with the statistics package SPSS ver. 25.0 (IBM, Armonk, NY, USA). A positive PCR test was set as an outcome variable and the independent variables were age, gender, health status, and infestation with ticks, fleas, or lice. The association of independent variables with outcome variables were evaluated by the chi-squared test, the Fisher’s exact test, and binary logistic regression analysis for calculation of the odds ratio. Differences were considered significant if the p-value was < 0.05.

## 5. Conclusions

All of the VBPs of dogs (*A. platys*, *B. vogeli*, *M. haemocanis*, and *B. henselae*), and in cats (*M. haemofelis*, *Candidatus* Mycoplasma haemominutum, *B. henselae*, and *A. platys*) are reported for the first time from these species in Saudi Arabia. The connection between these VBPs and their arthropod vectors in Saudi Arabia remains unknown and warrants further investigation. Climate appears to be the limiting factor affecting distribution of these pathogens, which may or may not be related to the distribution of their associated ectoparasite hosts. Considering that *B. henselae* and *A. platys* are known zoonotic pathogens and that dogs and cats pose human infection risks, effective ectoparasite control strategies are advocated. 

## Figures and Tables

**Table 1:** Prevalence of vector-borne pathogens in stray dogs (n = 70) and cats (n = 44) in the Asir Province, Saudi Arabia, using multiplex tandem real-time PCR. Dogs from Riyadh tested negative for all the pathogens (not included in the table).

Pathogen	No. of Infected Dogs (%)	No. of Infected Cats (%)	Accession Numbers *
*Anaplasma platys*	40 (57.1%)	1 (2.27%)	MW199129
*Babesia vogeli*	21 (30%)	0	MW204836
*Bartonella henselae*	1 (1.4%)	4 (9.1%)	MW208843
*Mycoplasma haemocanis/Mycoplasma haemofelis*	11 (15.7%)	6 (13.63%)	MW280822–MW280824
*Candidatus* Mycoplasma haemominutum	0	6 (13.63%)	ns

* Primers are listed in Section 4.6. Specific pathogens’ detection and sequencing; ns = not sequenced.

**Table 2:** Characteristics of 188 stray dogs and cats for study of the vector-borne pathogens in the Asir and Riyadh Provinces, Saudi Arabia.

Variable	Asir Province		Riyadh Province
	Dogs (*n* = 70)	Cats (*n* = 44)	Dogs (*n* = 74)
Age (years)			
≤1	19 (27.1%)	24 (54.6%)	17 (23%)
2–3	22 (31.4%)	10 (22.7%)	19 (25.7%)
4–5	8 (11.4%)	0 (0.0%)	10 (13.5%)
≥6	21 (30.0%)	10 (22.7)	28 (37.8%)
Sex			
male	38 (54.3%)	28 (63.6%)	42 (56.8%)
female	32 (45.7%)	16 (36.4%)	32 (43.2%)
Health status			
symptomatic	7 (10.0%)	5 (11.4%)	7 (9.5%)
asymptomatic	63 (90.0%)	39 (88.6%)	67 (90.5%)
Infestation with ectoparasites			
ticks	27 (38.6%)	11 (25.0%)	18 (24.3%)
fleas	22 (31.4%)	11 (25.0%)	29 (39.2%)
lice	6 (8.6%)	10 (22.7%)	15 (20.3%)

## Data Availability

Data sharing is not applicable to this article.

## Supplementary Materials

The following are available online at https://www.mdpi.com/2076-0817/10/1/25/s1, Table S1: Symptoms of dogs and cats from the Asir Province, Saudi Arabia, infected with VBPs detected via multiplex tandem real-time PCR.
