# Supplementary material for: Molecular Survey of Vector-Borne Pathogens of Dogs and Cats in Two Regions of Saudi Arabia
Source: Pathogens. 2020 Dec 31;10(1):25. doi: 10.3390/pathogens10010025 (PMC7823254; doi:10.3390/pathogens10010025)
Supplement: Supplementary file 1 [file pathogens-10-00025-s001.pdf]

Supplementary Table 1. Symptoms of dogs and cats from Asir province, Saudi Arabia, infected with VBPs detected via real-time Multiplex-Tandem PCR.

ID	Pathogens Detected	Fever	Anorexia	Emaciation	Colic	Red eyes
**Dog 5**	*Anaplasma platys, Babesia vogeli,*	+	+	-	+	-
**Dog 15**	*Anaplasma platys, Mycoplasma haemocanis*	-	-	+	-	+
**Dog 18**	*Anaplasma platys*	+	+	-	-	+
**Dog 39**	*Anaplasma platys, Babesia vogeli, Mycoplasma haemocanis*	+	-	-	+	+
**Dog 50**	*Anaplasma platys, Mycoplasma haemocanis*	+	+	+	-	-
**Dog 53**	*Anaplasma platys, Babesia vogeli, Mycoplasma haemocanis*	+	+	+	-	-
**Cat 5**	*Mycoplasma haemofelis, Candidatus* Mycoplasma haemominutum	+	+	-	-	+
**Cat 7**	*Candidatus* Mycoplasma haemominutum	-	-	+	+	-
**Cat 27**	*Mycoplasma haemofelis, Candidatus* Mycoplasma haemominutum	+	+	-	-	-
